# Pulse pressure as a predictor of Alzheimer’s disease biomarkers and cognitive decline: The moderating role of APOE ε4

**DOI:** 10.1016/j.tjpad.2025.100363

**Published:** 2025-09-03

**Authors:** Joon Hyung Jung, Nayeong Kong, Seunghoon Lee

**Affiliations:** a Department of Psychiatry, College of Medicine, Chungbuk National University, 1 Chungdae-ro, Seowon-gu, Cheongju, Republic of Korea, 28644; b Department of Psychiatry, Keimyung University Dongsan Hospital, 1035, Dalgubeol-daero, Dalseo-gu, Daegu, Republic of Korea, 42601; c Department of Psychiatry, Myongji Hospital, Hanyang University College of Medicine, 55 Hwasu-ro, Goyang, Republic of Korea, 10475

**Keywords:** Pulse pressure, Arterial stiffness, Alzheimer's disease, Amyloid-beta (Aβ), Tau, Apolipoprotein E ε4

## Abstract

**Background:**

Elevated pulse pressure (PP), indicative of arterial stiffness, has been implicated in cognitive impairment and Alzheimer’s disease (AD) pathology. However, its role in preclinical AD and interactions with genetic risk factors like apolipoprotein E ε4 (APOE4) remain unclear.

**Objectives:**

To investigate the association between baseline PP and AD biomarkers (amyloid-beta (Aβ) and tau) and cognitive decline, and to determine whether APOE4 carrier status moderates these relationships.

**Design:**

Prospective cohort study and secondary analysis of the Anti-Amyloid Treatment in Asymptomatic Alzheimer’s (A4) randomized clinical trial

**Setting:**

Multicenter observational cohort and randomized clinical trial conducted at 67 sites across the United States, Canada, Australia, and Japan.

**Participants:**

This study included 1690 cognitively unimpaired older adults from the A4 and Longitudinal Evaluation of Amyloid Risk and Neurodegeneration (LEARN) studies. Participants underwent baseline PP assessment, Aβ and tau PET imaging, and cognitive testing with longitudinal follow-up over 240 weeks.

**Measurements:**

Blood pressure was measured at baseline, with PP calculated as the difference between systolic and diastolic pressures. AD pathologies were assessed through Aβ PET imaging using 18F-Florbetapir, and regional tau deposition in inferior temporal and meta-temporal regions using 18F-Flortaucipir PET imaging. Cognitive performance was measured using the Preclinical Alzheimer Cognitive Composite (PACC).

**Results:**

Higher baseline PP was significantly associated with increased Aβ (β = 0.078; *p* = 0.001), inferior temporal tau (β = 0.110; *p* = 0.032), and meta-temporal tau deposition (β = 0.116; *p* = 0.022). In longitudinal analyses, elevated PP predicted greater decline in PACC scores (β = −0.020; *p* < 0.001). APOE4 status moderated these associations, with significant effects of PP on tau deposition and cognitive decline observed exclusively among APOE4 carriers. Mediation analysis indicated that tau deposition significantly mediated the association between PP and cognitive decline (indirect effect β = −0.068; 95 % CI [−0.126, −0.011]).

**Conclusions:**

Elevated PP is associated with increased AD biomarker burden and accelerated cognitive decline in cognitively unimpaired older adults, particularly among APOE4 carriers. Our study suggests that arterial stiffness may contribute to AD pathogenesis and progression via tau pathology. These results highlight the potential of vascular health management as an early intervention target for AD prevention, especially in genetically at-risk populations.

## Highlights

• Elevated pulse pressure (PP) is associated with increased amyloid-beta (Aβ) and tau deposition in the brain in a large cohort of cognitively unimpaired individuals.
• APOE ε4 moderates the relationship between PP and tau deposition.
• Higher PP is associated with worse cognitive performance, as indicated by lower scores on the Preclinical Alzheimer Cognitive Composite (PACC).
• Longitudinally, higher PP predicts a faster decline in cognitive function, particularly in APOE ε4 carriers.
• The association between PP and cognitive decline is mediated by tau deposition.

## 1 Introduction

Alzheimer’s disease (AD), the most common cause of dementia, is characterized by the progressive accumulation of amyloid-beta (Aβ) plaques and tau neurofibrillary tangles, coupled with neuronal loss and cognitive decline [1,2].

Vascular dysfunction, including chronic hypertension and increased arterial stiffness, is increasingly being recognized as a critical contributor to AD pathology [3,4]. Among various vascular risk factors, elevated pulse pressure (PP) has been identified as an accessible surrogate marker of arterial stiffness and overall vascular health [5,6]. Elevated PP has further been associated with an increased risk of cardiovascular and cerebrovascular diseases, which exacerbate cognitive decline and dementia [[7], [8], [9], [10]]. Regarding AD-related pathology, several studies have revealed significant associations of arterial stiffness with brain Aβ deposition [[11], [12], [13]]. Moreover, elevated PP has been associated with higher levels of phosphorylated tau and total tau in the cerebrospinal fluid (CSF), thereby indicating a potential mechanistic link between vascular dysfunction and neurodegeneration [4,14,15].

The apolipoprotein E ε4 (APOE4) allele, a well-established genetic risk factor for AD, is associated with vascular damage as well as an increased risk of cardiovascular small vessel disease [[16], [17], [18]]. Emerging evidence suggests that APOE4 may interact with vascular risk factors to further exacerbate AD pathology and cognitive decline [[19], [20], [21], [22]]. In a longitudinal study, elevated PP was associated with greater episodic memory decline, with the strongest effects observed among individuals carrying the APOE4 allele [19]. Similarly, midlife vascular risk factors have been shown to predict greater cognitive decline in APOE4 carriers decades later [20]. At the biomarker level, the Framingham Heart Study found that APOE4 status modified the effect of cardiovascular risk on tau deposition, as measured by tau PET, in cognitively normal individuals [22]. Therefore, understanding how PP interacts with APOE4 to influence AD pathology and cognition is crucial. However, these effects remain largely unexplored.

In this context, the present study aims to elucidate the relationship between baseline PP and key AD biomarkers, including both Aβ and tau deposition, as well as longitudinal changes in cognitive function. Utilizing data from the Anti-Amyloid Treatment in Asymptomatic Alzheimer’s (A4) and Longitudinal Evaluation of Amyloid Risk and Neurodegeneration (LEARN) studies, we hypothesized that elevated PP levels are associated with increased AD pathology and accelerated cognitive decline, particularly in APOE4 carriers.

## 2 Methods

### 2.1 Participants

A total of 1690 individuals from the A4 and LEARN studies were enrolled in this study (data accessed in November 2024). The A4 study is a double-blind clinical trial in a group of Aβ-positive, cognitively unimpaired older participants to evaluate the effect of the monoclonal antibody solanezumab. The trial was conducted at 67 sites in United States, Australia, Canada, and Japan, and the drug was provided by Eli Lilly [23]. This double-blind study was conducted over 240 weeks. The LEARN study included Aβ-negative participants in the A4 study screening who chose to participate and were assessed for cognitive changes in a manner similar to the A4 participants.

Participants in the A4 study were aged 65 to 85 years and had preclinical AD, defined by the following criteria: (i) Mini-Mental State Examination (MMSE) score of 27 to 30 for those with higher education (13 or more years) or 25 to 30 for those with lower education (12 or fewer years); (ii) Wechsler memory scale logical memory test part IIa score of 8 to 15 for participants with higher education or 6 to 13 for participants with lower education; (iii) A global CDR score of 0; and (iv) 18F-Florbetapir PET screening scans indicating brain Aβ pathology. Participants were excluded if they had (i) a screening MRI showing more than four ARIA-H (amyloid-related imaging abnormalities-hemorrhage) or any ARIA-E (edema/effusion), (ii) major or unstable illnesses (including unstable ischemic cardiovascular disease), or (iii) those requiring the use of excluded medications. The studies were conducted under the Declaration of Helsinki and the International Conference on Harmonization Good Clinical Practice (ICH-GCP) guidelines. The study protocols were approved by independent ethics committees or institutional review boards at each site. Written informed consent was obtained from all participants.

### 2.2 Measurement of blood pressure

Blood pressure measured at the baseline visit was used for this study. Measurements were taken in the sitting position. PP was calculated as systolic blood pressure minus diastolic blood pressure, and mean arterial pressure (MAP) was calculated as one-third of the systolic pressure plus two-thirds of the diastolic pressure. Participants with PP values outside the mean ± 3 standard deviations were excluded from the study (*n* = 6).

### 2.3 Measurement of AD pathologies

Aβ deposition was measured using 18F-Florbetapir PET. Global Aβ deposition was calculated as the mean standardized uptake value ratio (SUVR) across the regions of interest (ROIs), including the frontal, cingulate, lateral parietal, and lateral temporal cortices with a whole cerebellar reference region. Participants with a global Aβ deposition greater than 1.15 were classified as Aβ-positive. For participants with global Aβ deposition between 1.10 and 1.15, Aβ positivity was determined by consensus of two independent readers who confirmed a positive visual read; otherwise, participants were classified as Aβ-negative [24]. Tau deposition was measured in a subset of the participants (*n* = 440) using 18F-Flortaucipir PET. The SUVR was calculated using the cerebellar cortex as a reference. Two ROIs were defined in the study: an inferior temporal ROI and a meta-temporal ROI. The inferior temporal ROI was selected because the inferior temporal cortex serves as a proxy region for early neocortical tau spread [25,26]. The meta-temporal ROI was defined as the mean SUVR of the entorhinal cortex, amygdala, parahippocampal gyrus, fusiform gyrus, inferior temporal cortex, and middle temporal cortex, capturing a broad area of age-related and AD-related tau deposition [27].

### 2.4 Cognitive measures

Cognitive function was assessed using the Preclinical Alzheimer Cognitive Composite (PACC), a scale designed to measure amyloid-related cognitive decline in unimpaired populations [28]. The PACC comprises four components: the total score on the Free and Cued Selective Reminding Test (FCSRT), delayed paragraph recall on the Logical Memory IIa test from the Wechsler Memory Scale, Digit Symbol Substitution Test from the Wechsler Adult Intelligence Scale-Revised, and total MMSE score. Each component score was converted into a z-score by subtracting the baseline mean and dividing by the baseline standard deviation. The PACC score is defined as the sum of these z-scores, with negative scores indicating poor cognitive performance. Testing was conducted by certified psychometrists blinded to the trial-group assignments and adverse events.

### 2.5 Statistical analysis

Demographic and clinical variables between participants in the A4 and LEARN studies were compared using either chi-squared tests or independent *t*-tests. Cross-sectional analyses were performed using multiple linear regression models. In Model 1, global Aβ deposition, meta-temporal tau deposition, inferior temporal tau deposition, and PACC were set as dependent variables. PP was the independent variable, and covariates included age, sex, years of education, and APOE4 carrier status (carrier vs. non-carrier). Model 2 additionally adjusted for body mass index (BMI), smoking status (never vs. ever smoker), and MAP, in addition to the Model 1 covariates. Analyses were performed for the entire cohort and for the A4 subgroup. Interaction effects between PP and APOE4 carrier status were analyzed by including an interaction term (PP × APOE4 carrier status). If significant interactions were detected, subgroup analyses were performed, stratified by APOE4 carrier status.

For longitudinal analysis, linear mixed-effects models (LMMs) with random intercepts and slopes were used to evaluate the relationship between PP and cognitive change. In these models, PACC was the dependent variable, with PP, time (years from baseline), and their interaction as fixed effects. Covariates included age, sex, APOE4 carrier status, and years of education. Model 2 further adjusted for BMI, smoking status, and MAP. These analyses were conducted for the entire cohort and the A4 subgroup separately. To examine the moderating effects of APOE4 carrier status on cognitive change, a three-way interaction term (PP × time × APOE4 carrier status) was added to the Model 2. If interaction effects were significant, subgroup analyses stratified by APOE4 carrier status were conducted.

Mediation analysis was conducted to investigate the direct and indirect effects of PP on longitudinal cognitive decline using tau deposition as a mediator. The PACC slope for each participant was calculated using LMMs, with time (years from baseline) as the independent variable and PACC as the dependent variable. The mediation model specified PP as the independent variable, PACC slope as the dependent variable, and meta-temporal tau deposition as the mediator, adjusting for age, sex, years of education, and APOE4 carrier status. Bootstrap confidence intervals (CIs) from 10,000 bootstrap samples were used to estimate the indirect effects, with CIs not containing zero considered statistically significant. All statistical analyses were performed using R (version 4.3.0) and Process Macro for R (version 4.2), and statistical significance was set at p-values < 0.05. Standardized coefficients and their 95 % CIs were reported when applicable. For analyses involving longitudinal cognitive changes, the study group (Placebo, Solanezumab, and LEARN) was additionally adjusted for in the sensitivity analyses.

## 3 Results

### 3.1 Participant characteristics

A total of 1690 participants were included in the baseline evaluation, with a mean (SD) age of 71.5 (4.7) years; 1014 (60.0 %) were female. The detailed demographic and clinical characteristics of the participants are summarized in Table 1. Compared to the LEARN cohort, participants in the A4 cohort were older, more likely to be APOE4 carriers, had higher PP, and showed higher levels of Aβ and meta-temporal tau deposition (all *p* < 0.05).

**Table 1:** Demographic and clinical characteristics of the study participants.

	A4 (*n* = 1158)	LEARN (*n* = 532)	Total (*n* = 1690)	*p* value
Age, yr	71.9 (4.8)	70.5 (4.3)	71.5 (4.7)	< 0.001
Sex				0.445
Female	688 (59.4)	326 (61.3)	1014 (60.0)	
Male	470 (40.6)	206 (38.7)	676 (40.0)	
Education, yr	16.6 (2.8)	16.8 (2.6)	16.6 (2.7)	0.121
APOE4 carriers,	684 (59.1)	122 (22.9)	806 (47.7)	< 0.001
Pulse pressure, mmHg	58.7 (13.6)	56.5 (13.0)	58.0 (13.5)	0.002
Mean arterial pressure, mmHg	93.8 (9.9)	94.8 (9.3)	94.1 (9.7)	0.065
BMI, kg/m^2^	27.4 (5.1)	27.5 (4.9)	27.4 (5.0)	0.538
Ever smoker	20 (1.7)	3.0 (0.6)	23.0 (1.4)	0.056
Global Aβ deposition, SUVR	1.33 (0.18)	0.99 (0.07)	1.22 (0.22)	< 0.001
Aβ Centiloid	66.1 (32.9)	4.3 (12.6)	46.7 (40.2)	< 0.001
Meta-temporal tau deposition, SUVR (*n* = 440)	1.44 (0.22)	1.31 (0.09)	1.4 (0.2)	< 0.001
PACC	0.0 (2.7)	0.8 (2.3)	0.3 (2.6)	< 0.001
MMSE	28.8 (1.3)	29.0 (1.2)	28.9 (1.2)	< 0.001

* Data are presented as either means (standard deviations) or n ( %).

Abbreviations: A4, Anti-Amyloid Treatment in Asymptomatic Alzheimer’s; Aβ, amyloid-beta; APOE4, apolipoprotein E ε4; BMI, body mass index; LEARN, Longitudinal Evaluation of Amyloid Risk and Neurodegeneration; MMSE, Mini-Mental State Examination; PACC, Preclinical Alzheimer Cognitive Composite; SUVR, standardized uptake value ratio.

### 3.2 Association between baseline pulse pressure and AD biomarkers

**Fig. 1:**
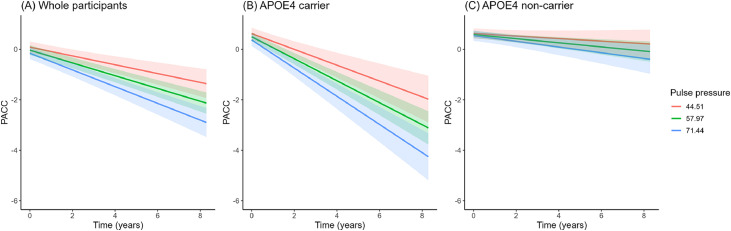
Association between pulse pressure and AD pathologies. (A) Meta-temporal tau deposition, (B) Global Aβ deposition, (C) Interaction effects of APOE4 carrier status on meta-temporal tau deposition. Linear regression plots with 95% confidence intervals. Abbreviations: Aβ, amyloid-beta; AD, Alzheimer’s disease; APOE4, apolipoprotein E ε4.

Baseline PP was positively associated with inferior temporal tau (β = 0.110, 95 % CI [0.010, 0.211], *p* = 0.032), meta-temporal tau deposition (β = 0.116, 95 % CI [0.017, 0.215], *p* = 0.022), and global Aβ deposition (β = 0.078, 95 % CI [0.032, 0.124], *p* = 0.001) after adjusting for age, sex, education, APOE4 carrier status, BMI, smoking and MAP (Table 2 and Fig. 1). Analyses restricted to the Aβ-positive subgroup (A4 study) yielded similar results for biomarkers, except for those related to global Aβ deposition (β = 0.054, 95 % CI [−0.005, 0.114], *p* = 0.074) (Supplementary Table 1).

**Table 2:** Association of the baseline pulse pressure with AD biomarkers in whole participants (A4 + Learn).

	Standardized Estimate (95 % CI)	*t* value	*p* value
Dependent variable: inferior temporal tau			
Model 1*	0.120 (0.027, 0.213)	2.529	0.012
Model 2^†^	0.110 (0.010, 0.211)	2.152	0.032
Dependent variable: meta-temporal tau			
Model 1*	0.108 (0.017, 0.200)	2.327	0.020
Model 2^†^	0.116 (0.017, 0.215)	2.297	0.022
Dependent variable: global Aβ deposition			
Model 1*	0.057 (0.013, 0.100)	2.547	0.011
Model 2^†^	0.078 (0.032, 0.124)	3.317	0.001

Abbreviations: A4, Anti-Amyloid Treatment in Asymptomatic Alzheimer’s; Aβ, amyloid-beta; APOE4, apolipoprotein E ε4; BMI, body mass index; CI, confidence intervals; LEARN, Longitudinal Evaluation of Amyloid Risk and Neurodegeneration.

⁎ Adjusted for age, sex, years of education andAPOE4 status^†^ Adjusted for age, sex, years of education, APOE4 status, body mass index, smoking status and mean arterial pressure.

### 3.3 Association between baseline pulse pressure and cross-sectional/longitudinal cognition

No significant association was observed between PP and baseline PACC scores in the total cohort (β = −0.046, 95 % CI [−0.093, 0.0001], *p* = 0.051); however, PP was negatively associated with baseline PACC in the Aβ-positive group (β = −0.070, 95 % CI [−0.127, −0.014], *p* = 0.015) (Supplementary Table 2 and Supplementary Table 3).

**Fig. 2:**
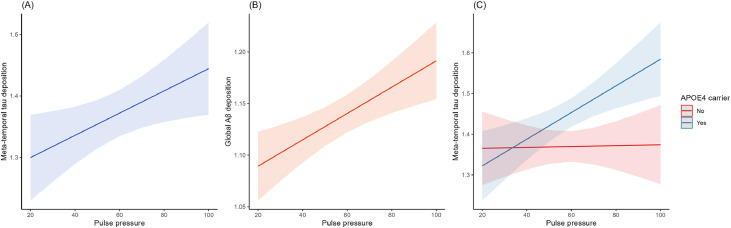
Association of pulse pressure with longitudinal cognitive changes. (A) Whole participants, (B) APOE4 carriers, (C) APOE4 non-carriers. Predicted cognitive function at mean pulse pressure, mean + 1 SD, and mean − 1 SD, derived from linear mixed models with 95 % confidence intervals. Abbreviations: APOE4, apolipoprotein E ε4; PACC, Preclinical Alzheimer Cognitive Composite; SD, standard deviation.

In longitudinal analyses, the interaction between PP and time was significantly associated with PACC scores (β = −0.020, 95 % CI [−0.031, −0.008], *p* < 0.001) (Supplementary Table 2). This finding indicates that a higher PP was associated with greater cognitive decline (Fig. 2). The same analyses restricted to the Aβ-positive subgroup revealed similar results (Supplementary Table 3).

### 3.4 Moderating effect of APOE4 on the association between pulse pressure, AD biomarkers, and baseline cognition

The APOE4 carrier status significantly moderated the association between PP and tau deposition in the inferior temporal region (*p* = 0.016) and meta-temporal tau deposition (*p* = 0.026). Subgroup analyses revealed that PP was significantly associated with increased tau deposition in APOE4 carriers (inferior temporal: β = 0.180, 95 % CI [0.041, 0.319], *p* = 0.011; meta-temporal tau: β = 0.193, 95 % CI [0.055, 0.331], *p* = 0.006) but not in non-carriers (Table 3). No significant interaction effects were observed for global Aβ deposition and PACC.

**Table 3:** Interaction analyses of APOE4 carrier status in the relationship between pulse pressure, AD biomarkers, and baseline cognition, with subgroup analyses.

Dependent variable	Interaction analysis	P-value	Subgroup analysis	Standardized estimate (95 % CI)	p-value
Inferior temporal tau	PP × APOE4	0.016			
			PP (APOE4 carrier)	0.180 (0.041, 0.319)	0.011
			PP (APOE4 non-carrier)	0.010 (−0.141, 0.162)	0.893
Meta-temporal tau	PP × APOE4	0.026			
			PP (APOE4 carrier)	0.193 (0.056, 0.331)	0.006
			PP (APOE4 non-carrier)	0.010 (−0.140, 0.159)	0.898
Aβ	PP × APOE4	0.163			
PACC	PP × APOE4	0.335			

Abbreviations: Aβ, amyloid-beta; AD, Alzheimer’s disease; APOE4, apolipoprotein E ε4; CI, confidence intervals; PACC, Preclinical Alzheimer Cognitive Composite; PP, pulse pressure.

Linear regression analyses were performed, controlling for age, sex, years of education, body mass index, smoking status, and mean arterial pressure. Subgroup analyses were conducted when the interaction analysis p-value was less than 0.05.

### 3.5 Moderating effect of APOE4 on the association between pulse pressure and longitudinal cognition

A significant interaction between PP, time, and APOE4 status was identified for the longitudinal changes in PACC scores (*p* = 0.049). Subgroup analyses revealed that a higher PP was associated with greater cognitive decline over time in APOE4 carriers (β = −0.027, 95 % CI [−0.044, −0.011], *p* = 0.001); however, no significant association was observed in non-carriers (Supplementary Table 4 and Fig. 2).

### 3.6 Mediation analysis between pulse pressure and longitudinal cognition

Mediation analysis revealed that tau deposition mediated the effects of PP on PACC changes. The indirect effect was significant (β = −0.068, 95 % CI [−0.126, −0.011]), indicating that PP indirectly influenced PACC changes through tau deposition; however, the direct effect of PP on PACC change was not significant (β = −0.034; *p* = 0.374) (Supplementary Figure 1).

### 3.7 Sensitivity analyses

Sensitivity analyses were conducted for longitudinal cognitive changes, with additional adjustments made for the study groups (Placebo, Solanezumab, and LEARN). These analyses yielded results consistent with the primary analyses.

## 4 Discussion

This study provides evidence that elevated PP, a marker of arterial stiffness, is significantly associated with increased Aβ and tau deposition, as well as accelerated cognitive decline in a large cohort of cognitively unimpaired older adults. Notably, these associations were particularly pronounced in individuals harboring the APOE4 allele. Furthermore, our mediation analysis suggested that tau deposition plays a crucial role in mediating the relationship between PP and cognitive decline.

Our findings build upon and extend previous research demonstrating associations between PP, arterial stiffness, and AD pathology [14,15]. For instance, a study using ADNI data observed higher PP in CSF p-tau positive participants, while PP was negatively associated with Αβ1–42 in participants over 80 years old [14]. Similarly, a study from the Framingham cohort found that aortic stiffness and pressure pulsatility were associated with tau burden in the rhinal and entorhinal regions, which are critical for early tau accumulation in AD [29]. Our study expands upon these findings by demonstrating a robust association between PP and both Aβ and tau deposition in a large, cognitively unimpaired cohort. While prior studies have reported mixed results regarding the effects of PP or arterial stiffness on Aβ deposition [[11], [12], [13], [14], [15]], our observation of a significant association may be attributed to the larger sample size, as well as the inclusion of a high proportion of Aβ-positive participants. Additionally, we identified significant associations between PP and tau deposition in the inferior temporal and meta-temporal ROIs, both of which are associated with cognitive decline and progression in preclinical AD [[30], [31], [32], [33]].

Elevated PP may increase Aβ and tau deposition through several mechanisms. First, elevated PP is directly correlated with cerebral blood flow pulsatility and can reduce cerebral blood flow [34]. Altered cerebral blood flow may potentially hinder the clearance of Aβ from the brain and promote tau phosphorylation [[35], [36], [37], [38]]. Second, disruption of the neurovascular unit (NVU) by elevated PP can facilitate the entry of toxic materials into the brain, leading to increased microglial activity and neuroinflammation [39,40]. This heightened neuroinflammation and microglial hyperactivity are robustly associated with the progression of AD pathology [41]. Our previous study also suggested that elevated PP is related to presynaptic dysfunction, which can contribute to tau deposition [42].

In the longitudinal analyses, elevated PP was associated with a greater decline in cognitive performance over time, as measured by the PACC. This association remained significant even after adjusting for covariates, such as age, education, and other vascular risks. Our mediation analysis revealed that tau mediates the association between PP and cognitive decline. These results indicate that elevated PP indirectly influences cognitive decline by promoting tau accumulation. While prior studies have reported associations between PP and tau deposition or between PP and cognitive decline, our study is one of the first to link these associations. This finding aligns with the current understanding of AD, in which Aβ precedes tau pathology, and the propagation of tau pathology across the brain cortex causes neurodegeneration and cognitive decline [43].

Notably, the association among PP, tau deposition, and longitudinal cognitive decline was particularly pronounced in APOE4 carriers, suggesting that genetic susceptibility to AD may amplify the negative effects of vascular dysfunction on cognitive outcomes. These findings align with those of prior studies involving non-demented older adults that reported links between arterial stiffness, poorer executive function, and episodic memory, particularly in APOE4 carriers [44]. APOE4 is expressed in astrocytes, microglia, and vascular cells, influencing lipid metabolism, Aβ clearance, and neuroinflammation [[45], [46], [47], [48]]. In the presence of elevated PP, APOE4 carriers may experience amplified NVU disruption, resulting in a greater deposition of tau. Additionally, APOE4 has been shown to enhance tau pathology independently of Aβ via microglial activation and impaired lipid homeostasis [[49], [50], [51]]. It is also associated with microvascular dysfunction and blood-brain barrier breakdown, which may potentiate cerebral hypoperfusion and oxidative stress [40,52]. At the cellular level, APOE4 status has been linked to lipid droplet accumulation in microglia [45] and enhanced ferroptosis—a regulated form of cell death driven by iron-dependent lipid peroxidation [46,48]. Elevated PP may further exacerbate these APOE4-driven mechanisms by enhancing oxidative stress, impairing glial function, and activating ferroptotic pathways, potentially leading to tau hyperphosphorylation and propagation. These interactions may help explain the heightened vulnerability of APOE4 carriers to the deleterious effects of vascular dysfunction, which could amplify the cognitive effects of elevated PP [52].

Our study has several strengths, including the use of a large, well-characterized cohort from the A4 and LEARN studies, which enabled a robust analysis. The inclusion of individuals with both Aβ-positive and Aβ-negative, combined with rigorous longitudinal follow-up, facilitated a more comprehensive investigation of the effects of PP on preclinical AD.

However, this study has several limitations that need to be considered. First, PP was used as a surrogate marker for arterial stiffness. While PP is widely recognized for its simplicity and accessibility in reflecting vascular health, it is an indirect measure that can be influenced by factors such as cardiac output and peripheral vascular resistance [53]. The gold standard for assessing arterial stiffness is carotid-femoral pulse wave velocity, which has shown moderate correlations with PP in prior studies [[54], [55], [56]]. Future studies incorporating direct measures of arterial stiffness are warranted. Second, although our analyses adjusted for key confounders such as age, sex, education, MAP, and BMI, they did not account for unmeasured confounders, such as lifestyle variables and comorbidities, which may have influenced the observed associations. Specifically, previous studies have reported that midlife hypertension, physical activity, and diabetes are associated with both dementia onset and arterial stiffness, and may therefore serve as major confounding factors [[57], [58], [59]]. Nevertheless, our study benefited from a large, well-characterized sample, thereby reducing the likelihood of significant bias. Third, while our biomarker analyses were cross-sectional, limiting causal inference between PP, Aβ, and tau deposition, our mediation model incorporated longitudinal cognitive assessments. This design reduces reverse causation concerns; however, future studies with repeated biomarker measurements are required to confirm the temporal relationships.

In conclusion, the present study demonstrates a significant association between elevated PP and increased Aβ and tau deposition, as well as accelerated cognitive decline, in cognitively unimpaired older adults. This association was particularly pronounced in APOE4 carriers, highlighting the interplay between vascular risk and genetic susceptibility in AD pathogenesis. These findings suggest that PP monitoring and management could be prioritized in APOE4 carriers. Such an approach may help tailor vascular risk control interventions for individuals at highest genetic risk, thereby contributing to more personalized prevention strategies in preclinical AD.

## Funding

This research was supported by the Korea National Institute of Health (NIH) research project (project No. 2023-ER1003–02).

The A4 and LEARN Studies were supported by a public-private-philanthropic partnership which included funding from the National Institute of Aging - the National Institutes of Health (R01 AG063689, U19AG010483 and U24AG057437), Eli Lilly (also the supplier of active medication and placebo), the Alzheimer’s Association, the Accelerating Medicines Partnership through the Foundation for the National Institutes of Health, the GHR Foundation, the Davis Alzheimer Prevention Program, the Yugilbar Foundation, an anonymous foundation, and additional private donors to Brigham and Women’s Hospital, with in-kind support from Avid Radiopharmaceuticals, Cogstate, Albert Einstein College of Medicine and the Foundation for Neurologic Diseases.

## Declaration of generative AI and AI-Assisted technologies in the writing process

We confirm that we have not used any AI at all except for English editing.

## Data availability statement

Data analyzed in the study are available on the A4 study. (https://www.a4studydata.org/)

## CRediT authorship contribution statement

**Joon Hyung Jung:** Writing – review & editing, Writing – original draft, Methodology, Investigation, Formal analysis, Data curation, Conceptualization. **Nayeong Kong:** Writing – original draft, Methodology, Investigation, Formal analysis. **Seunghoon Lee:** Writing – review & editing, Writing – original draft, Project administration, Methodology, Investigation, Formal analysis, Conceptualization.

## Declaration of competing interest

The authors declare that they have no known competing financial interests or personal relationships that could have appeared to influence the work reported in this paper.

## Appendix Supplementary materials

Image, application 1
