# Supplementary material for: Pulse pressure as a predictor of Alzheimer’s disease biomarkers and cognitive decline: The moderating role of APOE ε4
Source: J Prev Alzheimers Dis. 2025 Sep 3;12(10):100363. doi: 10.1016/j.tjpad.2025.100363 (PMC12627889; doi:10.1016/j.tjpad.2025.100363)
Supplement: Supplementary file 1 [file mmc1.docx]

**Supplementary Materials**

**Supplementary Tables**

**Supplementary Table 1.** Association of the baseline pulse pressure with AD biomarkers in the A4 group

**Supplementary Table 2.** Association of the baseline pulse pressure with cross-sectional and longitudinal cognition in whole participants (A4 + LEARN)

**Supplementary Table 3.** Association of the baseline pulse pressure with cross-sectional and longitudinal cognition in the A4 group

**Supplementary Table 4.** Interaction analyses of Apolipoprotein E ε4 carrier status on the association between PP and longitudinal change in PACC with subgroup analyses

**Supplementary Figure**

**Supplementary Figure 1.** Mediation effects of meta-temporal tau deposition on the association between pulse pressure and cognitive changes

| **Supplementary Table 1.** Association of the baseline pulse pressure with AD biomarkers in the A4 group | | | |
| --- | --- | --- | --- |
|  | Standardized Estimate  (95% CI) | *t* value | *p* value |
| Dependent variable: inferior temporal tau |  |  |  |
| Model 1^*^ | 0.121 (0.022, 0.221) | 2.398 | 0.017 |
| Model 2^†^ | 0.103 (−0.004, 0.210) | 1.899 | 0.058 |
| Dependent variable: meta-temporal tau |  |  |  |
| Model 1^*^ | 0.111 (0.012, 0.209) | 2.214 | 0.027 |
| Model 2^†^ | 0.110 (0.005, 0.216) | 2.051 | 0.041 |
| Dependent variable: global Aβ deposition |  |  |  |
| Model 1^*^ | 0.035 (−0.021, 0.091) | 1.229 | 0.219 |
| Model 2^†^ | 0.054 (−0.005, 0.114) | 1.788 | 0.074 |
| Abbreviations: A4, Anti-Amyloid Treatment in Asymptomatic Alzheimer’s; Aβ, amyloid-beta; APOE4, apolipoprotein E ε4; BMI, body mass index; CI, confidence interval; LEARN, Longitudinal Evaluation of Amyloid Risk and Neurodegeneration.  ^*^ Adjusted for age, sex, years of education, APOE4 status  ^†^ Adjusted for age, sex, years of education, APOE4 status, body mass index, smoking status, and mean arterial pressure | | | |

| **Supplementary Table 2.** Association of the baseline pulse pressure with cross-sectional and longitudinal cognition in whole participants (A4 + LEARN) | | | | |
| --- | --- | --- | --- | --- |
| Dependent variable:  PACC | | Standardized Estimate  (95% CI) | *t* value | *p* value |
| Cross-sectional analysis | |  |  |  |
| Model 1^*^ | PP | −0.047 (−0.091, −0.004) | −2.124 | 0.034 |
| Model 2^†^ | PP | −0.046 (−0.093, 0.0001) | −1.956 | 0.051 |
| Longitudinal analysis | |  |  |  |
| Model 1^*^ | PP × time | −0.020 (−0.031, −0.008) | −3.412 | < 0.001 |
| Model 2^†^ | PP × time | −0.020 (−0.031, −0.008) | −3.418 | < 0.001 |
| Abbreviations: A4, Anti-Amyloid Treatment in Asymptomatic Alzheimer’s; APOE4, apolipoprotein E ε4; CI, confidence intervals; LEARN, Longitudinal Evaluation of Amyloid Risk and Neurodegeneration; PACC, Preclinical Alzheimer Cognitive Composite; PP, pulse pressure.  *Adjusted for age, sex, years of education, APOE4 status  †Adjusted for age, sex, years of education, APOE4 status, body mass index, smoking status and mean arterial pressure | | | | |

| **Supplementary Table 3.** Association of the baseline pulse pressure with cross-sectional and longitudinal cognition in the A4 group | | | | |
| --- | --- | --- | --- | --- |
| Dependent variable:  PACC | | Standardized Estimate (95% CI) | *t* value | *p* value |
| Cross-sectional analysis | |  |  |  |
| Model 1^*^ | PP | −0.065 (−0.118, −0.013) | −2.428 | 0.015 |
| Model 2^†^ | PP | −0.070 (−0.127, −0.014) | −2.443 | 0.015 |
| Longitudinal analysis | |  |  |  |
| Model 1^*^ | PP × time | −0.021 (−0.036, −0.007) | −2.997 | 0.003 |
| Model 2^†^ | PP × time | −0.022 (−0.036, −0.007) | −3.001 | 0.003 |
| Abbreviations: A4, Anti-Amyloid Treatment in Asymptomatic Alzheimer’s; APOE4, apolipoprotein E ε4; CI, confidence intervals; PACC, Preclinical Alzheimer Cognitive Composite; PP, pulse pressure.  *Adjusted for age, sex, years of education, APOE4 status  †Adjusted for age, sex, years of education, APOE4 status, body mass index, smoking status, and mean arterial pressure | | | | |

| **Supplementary Table 4.** Interaction analyses of APOE4 carrier status on the association between pulse pressure and longitudinal change in PACC with subgroup analyses | | | | | |
| --- | --- | --- | --- | --- | --- |
| Dependent variable | Interaction analysis | *p* value | Subgroup  analysis | Standardized Estimate  (95% CI) | *p* value |
| PACC | PP × APOE4 × time | 0.049 |  |  |  |
|  |  |  | PP × time (APOE4 carrier) | −0.027 (−0.044, −0.011) | 0.001 |
|  |  |  | PP × time (APOE4  non-carrier) | −0.010 (−0.024, 0.005) | 0.183 |
| Abbreviations: APOE4, apolipoprotein E ε4; CI, confidence intervals; PACC, Preclinical Alzheimer Cognitive Composite; PP, pulse pressure.  Linear mixed models were performed, controlling for age, sex, education, BMI, smoking status, and mean arterial pressure. Subgroup analyses were conducted when the interaction analysis p-value was less than 0.05. | | | | | |

**Supplementary Figure**

**
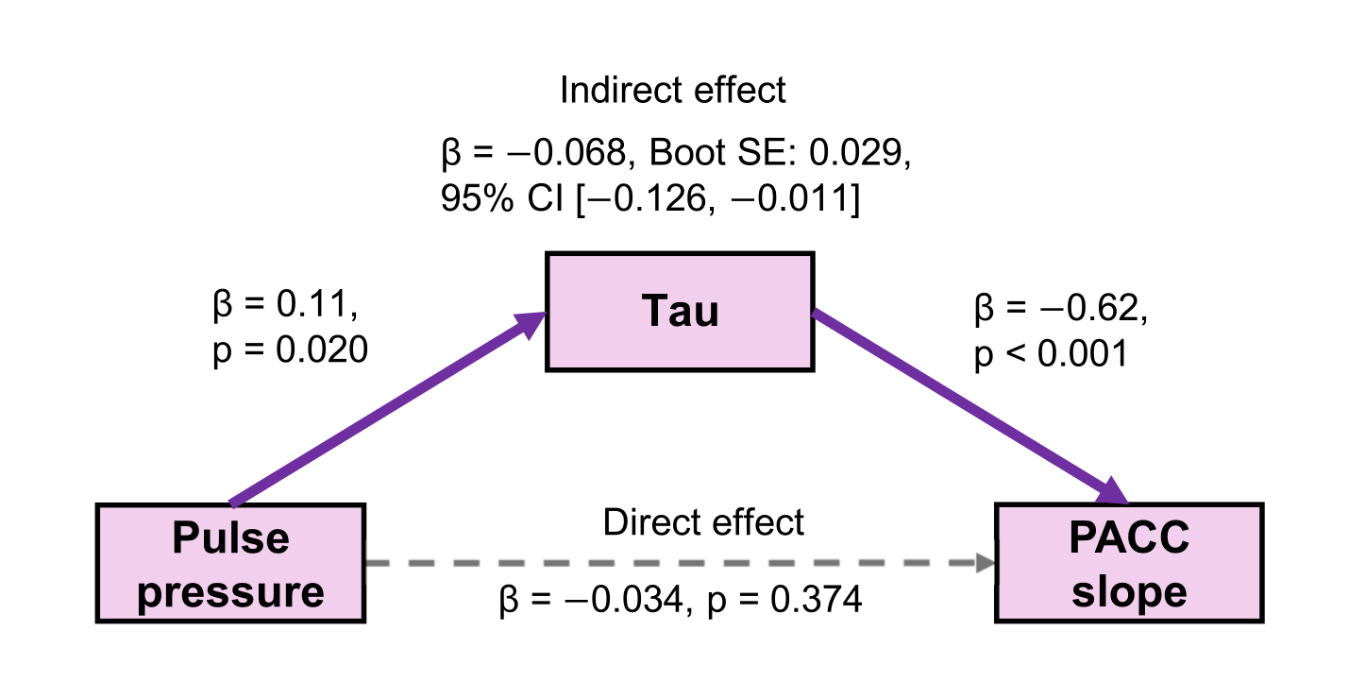
**

**Supplementary Figure 1.** Mediation effects of meta-temporal tau deposition on the association between pulse pressure and cognitive changes

Abbreviations: β, standardized estimate, PACC, Preclinical Alzheimer Cognitive Composite; Boot SE, Bootstrap standard error

The bold lines indicate p-values of < 0.05.
